# Investigation of the Impact of Dental Care via Composite Resin Restoration among Children with Attention Deficit Hyperactivity Disorder: A Registry-Based Nested Case–Control Study

**DOI:** 10.3390/healthcare9070803

**Published:** 2021-06-25

**Authors:** Chien-Jen Hu, Hui-Chieh Yu, Yu-Chao Chang

**Affiliations:** 1 School of Dentistry, Chung Shan Medical University, Taichung 402, Taiwan; stormmasakimou@gmail.com (C.-J.H.); yujessica7@gmail.com (H.-C.Y.); 2 Department of Dentistry, Chung Shan Medical University Hospital, Taichung 402, Taiwan

**Keywords:** attention deficit hyperactivity disorder, composite resin, nested case–control, children

## Abstract

Attention deficit hyperactivity disorder (ADHD) is one of the most common psychiatric conditions. Many studies have shown that exposure to low-dose bisphenol-A (BPA) can contribute to ADHD. The relationship between ADHD and composite resin is still unclear. We designed a nested case–control study to investigate the relationship between ADHD and composite resin restorations among children from the Taiwan’s National Health Insurance Research Database. We included 85,503 people whose birth years were between 1998 and 2005 and who received resin restoration from January 2000 to December 2008. A 1:2 matched control sample without ADHD was randomly selected from the database by propensity score matching based on birth year and sex. We identified 4984 individuals as ADHD patients. The average diagnostic age of ADHD was 7.45 years old. The patients who received composite resin restorations had higher risk of ADHD than those who had never received them (aOR (adjusted odds ratio) = 1.25; 95% CI (confidence interval) = 1.13–1.38). Males had a higher risk of ADHD (aOR = 1.29; 95% CI = 1.14–1.43). Taken together, this nested case–control study demonstrated a positive association between ADHD and dental care via composite resin restoration in Taiwanese children. Prospective clinical studies of the relationship between ADHD and composite resin are warranted.

## 1. Introduction

Attention deficit hyperactivity disorder (ADHD), characterized by developmentally inappropriate levels of hyperactivity, impulsivity, and inattention, is one of the most common childhood psychiatric conditions. Children are often diagnosed with ADHD during preschool years following symptoms such as frequent fidgeting or squirmy behaviors, an inability to follow instructions, and high distractibility. The consequences of ADHD can persist through adulthood if left untreated or uncontrolled. About 6.7% prevalence of ADHD was reported by US National Health Interview Survey from 1997 to 2000 [1]. In Taiwan, the prevalence of ADHD was estimated at approximately 9.9% [2]. The exact etiology of ADHD is still unclear. Despite heredity factors, it might be due to biological and environmental factors such as exposure to low-dose bisphenol-A (BPA). High urinary concentrations of BPA have been associated with ADHD occurrence in US children [3].

BPA is widely used to make polycarbonate plastics such as those in hard plastic baby, water bottles, and epoxy resins for dental sealants/resin-based composites. BPA is a kind of endocrine-disrupting chemical, which may affect brain development and increase the impairment of social communication in children [4]. Dental caries is the most common oral disease in children worldwide. Resin-based composite materials are the most popular filling materials for prepared cavity filling nowadays. The monomer bis-GMA can release BPA into the saliva, blood, and urine. Previous studies have shown that BPA levels were detectable after resin-based composite restoration placement [5,6,7].

Up to now, there has been no study examining the impact of dental care via composite resin restoration for ADHD. The aim of this study was to investigate the possible relationship between composite resin restoration and ADHD among children. Therefore, we designed a nested case–control study from the Taiwan’s National Health Insurance Research Database (NHIRD).

## 2. Materials and Methods

### 2.1. Study Population

The Longitudinal Health Insurance Database (LHID) 2010, a part of Taiwan’s NHIRD, was used for this study. Almost all of the Taiwanese population was enrolled in this compulsory National Health Insurance program. The LHID 2010 collected registration information and dental and medical data and contains 1 million beneficiaries randomly sampled from the 2010 registry of beneficiaries in the NHIRD. This study was approved by the Chung Shan Medical University Hospital Ethics Review Board. This report complies with STROBE (Strengthening the Reporting of Observational Studies in Epidemiology) guidelines for the observational studies.

### 2.2. Study Design and Sampled Individuals

**Figure 1:**
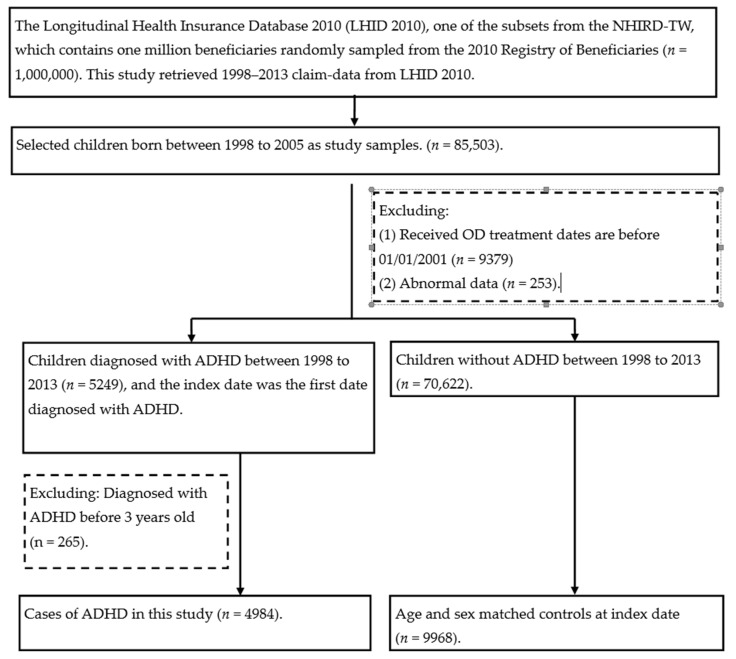
The flowchart of study sample selection from the National Health Insurance Research Database in Taiwan. NHIRD: Taiwan’s National Health Insurance Research Database.

Subjects of the study were children from the LHID 2010 whose birth years were between 1998 and 2005 and who received operative dentistry (OD) treatment between January 2000 and December 2008. The codes 89,004, 89,005, 89,008, 89,009, 89,010, 89,012, 89,104, 89,105, 89,108, 89,109, 89,110, and 89,112 for OD treatment indicate composite resin restoration. Patients were followed from the first date for composite resin restoration until 5 years after the date OD treatment was received (Figure 1).

The diagnosis of ADHD is according to ICD-9 codes 314.00, 314.01, and 314.9. Only two or more repeated diagnoses of ADHD in one year during 2005–2013 were considered to ensure accurate diagnostic criteria. The date on which each patient was first diagnosed with ADHD was defined as the index date. Matched controls (1:2) were randomly selected from the LHID 2010 by using propensity score matching based on both birth year and sex. Psychiatric comorbidities including autism, mental retardation, and developmental delay were assessed as the confounding factors in this study.

### 2.3. Statistical Analysis

All data analysis was performed using SAS 9.3 (SAS Institute, Cary, NC, USA). Statistical analyses of demographic characteristics and socioeconomic status of study subjects were performed using Student’s *t*-test for continuous variables and the chi-squared test for categorical variables. Conditional logistic regression was used to estimate crude and adjusted odds ratios (ORs) with a 95% confidence interval (CI) for the case group compared with the control group. In multivariate analysis, adjustments were made for age and sex.

## 3. Results

The characteristics and socioeconomic status of study subjects with and without ADHD are presented in Table 1. After matching, 14,952 subjects with birth years between 1998 and 2005 were included in this study. The number of males was higher than that of females (78.1% versus 21.9%). The average age of subjects with ADHD diagnosis was 7.45 years old. In addition, the ADHD group had more psychiatric comorbidities and a higher proportion of living in rural areas in comparison with the non-ADHD control group (*p* < 0.001).

Table 2 presents the relationship between composite resin restoration and ADHD. After adjusting for age, patients with a received composite resin restoration had a higher risk of ADHD than patients without one (aOR = 1.25; 95% CI = 1.13–1.38). The adjusted OR of composite resin restoration for ADHD was 1.14 (95% CI = 0.92–1.41) and 1.29 (95% CI = 1.14–1.43) for non-composite resin restorations for females and males, respectively.

The odds ratio for the number of composite resin restorations of those with a diagnosis of ADHD is shown in Table 3. In this study, the odds ratios of children with 1–3 times, 4–7 times, 8–11 times, and ≥1 2 times composite resin restorations were 1.19, 1.08, 1.10, and 1.24-fold risk of ADHD, respectively. However, children with 4–7 times and 8–11 times composite resin restorations did not reach the statistical significance. The results did not reveal a positive association with an increase in the number of restorations.

Table 4 illustrates the odds ratio for the time interval from the first composite resin restoration to the index date with ADHD. Logistic regression analysis showed that the risk of ADHD was 1.19-fold within 1 year after composite resin restoration (95% CI, 1.04–1.35) and 1.25-fold after 1 year (95% CI, 1.14–1.37) compared to those without composite resin restoration. In addition, there was also a positive association with an increased time interval up to 3 years from the first composite resin restoration.

## 4. Discussion

To the best of our knowledge, this nested case–control study first demonstrated the association between the composite resin restoration and ADHD among children. Males had a higher risk of ADHD. There was an increasing pattern in the prevalence of ADHD in Taiwan from 2000–2011 [8]. The female-to-male ratio of ADHD in Taiwan was reported at about 1:3.5 [9]. Some studies have indicated that males more easily suffer from ADHD than females [10,11]. We included 4984 ADHD patients in our study, with 1093 females (21.9%) and 3891 males (78.1%). It is similar to other studies showing a higher prevalence of ADHD among males than females. Consistently, a cross-sectional nationwide survey in US had reported that the higher urinary BPA concentrations were associated with ADHD and the associations were stronger in boys than in girls [3].

Despite the heritability factors of ADHD, environmental toxins may also affect childhood neurobehavioral outcomes such as BPA. As an endocrine disruptor, BPA may affect dopamine concentration at the dopaminergic neuron terminals and functionally impair dopamine receptors in ADHD patients [12]. When dopamine receptors impaired by BPA bring ADHD symptoms such as anxiety, hyperactivity, and attention [13].

Resin-based composite resin and dental sealants are the most commonly used materials in caries treatment and prevention, especially in children. Many studies have shown that BPA was detected after using resin-based composite for dental caries restoration [14,15,16,17,18]. This may be the reason why dental care via composite resin restoration is associated with ADHD. Therefore, the prevention of the occurrence of dental caries may not only provide a benefit for oral health but also for the prevention of ADHD.

In this study, we also evaluated the number of composite resin restoration and the time interval from the first composite resin restoration to the index date. There is no acceleration in ADHD patients where a higher number of composite resin restorations was present. However, the results show that the longer the duration of the composite resin restorations, the greater the risk of ADHD. The reason for this is not quite clear. Composite resin may wear out due to chewing, leading to BPA release into oral environments gradually. Taken together, further detailed studies need to evaluate the duration, number, size, and tooth surface of composite resin restorations in the pathophysiology of ADHD.

The possible confounders and other predictors of ADHD included a low birthweight, maternal smoking, alcohol/drug use, low birthweight, prematurity, toxic exposure, diet, and social/family environment [19]. In this study, we focused on childhood ADHD to reduce the multiple interactions of ADHD. With the nested case–control design, the time-dependent effect could be estimated. The use of a nationwide population-based database provided a sufficient sample size, generalizability, and statistical power to assess the association of dental care via composite resin restorations and children ADHD.

There are still some limitations in this study. First, there are multiple exposure sources of the human intake of BPA, including the atmosphere, water, effluent, food, drinking water, and dust [20]. However, no data were available to evaluate BPA levels and sources in the LHID 2010, nor were there data on the chemicals from dental composite resins, such as bis-GMA, UDMA, or TEGDMA. Second, the database does not contain information regarding the level of ADHD severity and the critical cause of ADHD. This analysis cannot confirm whether BPA in the etiology of ADHD occurrence was released from composite resin. Therefore, an evaluation of changed BPA levels in serum, blood, urine, and saliva from new composite resin restorations for the verification of ADHD still needs to be conducted. Third, since the treatment code of the NHI system represents the number of fillings in a tooth, the exact tooth surface, such as occlusal or buccal surface, could not be obtained from the NHIRD. The wear out effect of the composite resin could not be measured in the present study.

Our study demonstrated a positive association between composite restorations and ADHD among children. To focus on environmental toxins, dentists need to evaluate the potential harmful effects of composite-resin-based filling materials. During dental cavity restoration, exposure to unpolymerized dental resin materials must be controlled and limited to prevent the release of BPA after restoration. The development of a BPA-free composite resin as an alternative is encouraged. While BPA can directly contribute to ADHD, we should point out that dental composite resin is not the only route of BPA exposure in humans. Therefore, further studies are needed to confirm the causal relationship between dental care via composite resin restorations and ADHD among children.

## 5. Conclusions

There has hitherto been no report on the relationship between dental care via composite resin restorations and ADHD. Our findings demonstrated a positive association between composite resin restorations and ADHD among children. These associations were stronger in males than in females. Since composite resin remains a source of BPA exposure, other alternatives for restorative materials are suggested. Further studies are required to clarify the causal relationship between composite resin restorations and ADHD among children.

## Figures and Tables

**Table 1:** Demographic characteristics and socioeconomic status of study subjects.

	Total *n* = 14,952		With ADHD *n* = 4984		Without ADHD *n* = 9968		*p*
	*N*	%	*N*	%	*N*	%	
Composite resin treatment number	10,196	68.2	3500	70.2	6696	67.2	0.0002
Age at index date (Mean ± SD)			7.45 ± 2.20		7.45 ± 2.20		0.9946
Follow up time (Mean ± SD/years)			3.17 ± 2.15		3.15 ± 2.15		0.8596
Sex							1.00
female	3279	21.9	1093	21.9	2186	21.9	
male	11,673	78.1	3891	78.1	7782	78.1	
Comorbidity							
autism	430	2.9	392	7.8	38	0.4	<0.001
mental retardation	526	3.5	473	9.4	53	0.5	<0.001
developmental delay	1687	11.3	923	18.5	391	3.9	<0.001
Living district							<0.001
Taipei	5662	38.4	2245	46.2	3417	34.6	
Northern	2291	15.5	731	15.0	1560	15.8	
Central	2603	17.7	668	13.8	1935	19.6	
Southern	1907	12.9	490	10.1	1417	14.3	
Kao-Ping	1981	13.4	638	13.1	1343	13.6	
Eastern	304	2.06	87	1.79	217	2.2	
Urbanization level							<0.001
urban	11,415	77.4	3973	81.8	7442	75.3	
suburban	1229	8.33	336	6.9	893	9.03	
rural	2104	14.3	550	11.3	1554	15.7	

**Table 2:** Odds ratio for composite resin restorations of those with a diagnosis of ADHD.

		Total (*n* = 14,952)	Case (*n* = 4984)	Control (*n* = 9968)	Odds Ratio	95% CI
Without composite resin restoration		4756	3272	1484	1	
With composite resin restoration	total	10,196	3500	6696	1.25	1.13–1.38
	female	2294	772	1522	1.14	0.92–1.41
	male	7902	2728	5174	1.29	1.14–1.43

Abbreviations: OR, odds ratio; CI, confidence interval. Adjustment by age.

**Table 3:** Odds ratio for the number of composite resin restorations of those with a diagnosis of ADHD.

		Odds Ratio	95% CI
Without composite resin treatment		1	
With composite resin treatment	1–3 times	1.19	1.08–1.32
	4–7 times	1.08	0.97–1.02
	8–11 times	1.10	0.97–1.24
	≥12 times	1.24	1.12–1.38

Abbreviations: OR, odds ratio; CI, confidence interval. Adjustment by age and sex.

**Table 4:** Odds ratio for the time interval from the first composite resin restoration to the index date with ADHD.

		Total (*n* = 14,952)	Odds Ratio	95% CI
		*n*		
Without composite resin treatment		4756	1	--
With composite resin treatment	≤1 years	1639	1.19	1.04–1.35
	>1 years	8557	1.25	1.14–1.37
	≤3 years	5443	1.21	1.10–1.32
	>3 years	4753	1.30	1.16–1.50

Abbreviations: OR, odds ratio; CI, confidence interval. Adjustment by age and sex.

## Data Availability

Data sharing is not applicable.
